# Risk and protective factors for suicide: a populational case-control study, Brazil, 2019

**DOI:** 10.11606/s1518-8787.2023057004606

**Published:** 2023-03-29

**Authors:** Ana Maria Pita Ruiz, Mirla Randy Bravo Fernandez, Denis Satoshi Komoda, Carlos Alberto dos Santos Treichel, Ricardo Carlos Cordeiro

**Affiliations:** I Universidade Estadual de Campinas Faculdade de Ciências Médicas Campinas São Paulo Brasil Universidade Estadual de Campinas. Faculdade de Ciências Médicas. Campinas, São Paulo, Brasil

**Keywords:** Suicide, epidemiology, Protective Factors, Risk Factors, Case-Control Studies

## Abstract

**OBJETIVE:**

To estimate risk and protection factors associated with suicide in Campinas, Brazil, in 2019.

**METHODS:**

This is a populational case-control study analyzing 83 cases of suicide that occurred in 2019 in Campinas, a Brazilian city with about 1.2 million inhabitants. Controls were composed of 716 inhabitants. An adjusted multiple logistic regression was used. Cases and controls were the dichotomous response variables. Sociodemographic and behavioral variables were the predictor variables.

**RESULTS:**

The categories which presented higher risk of suicide were: males [OR = 5.26 (p < 0.001)]; people aged 10–29 years [OR = 5.88 (p = 0.002)]; individuals without paid work [OR = 3.06 (p = 0.013)]; individuals presenting problematic use of alcohol [OR = 33.12 (p < 0.001)] and cocaine [14.59 (p < 0.007)]; and people with disabilities [OR = 3.72 (p < 0.001)]. Moreover, the perception of fear was associated with reduced suicide risk [OR = 0.19 (p = 0.015)]. Higher district HDI levels also showed a 4% decrease in risk for each 0.01 increase in district HDI levels [OR = 0.02 (p = 0.008)].

**CONCLUSIONS:**

This study evidenced the association between sociodemographic and behavioral variables and suicide. It also emphasized the complexity in the dynamics between personal, social, and economic factors to this external cause of death.

## INTRODUCTION

Suicide is a major public health issue in Brazil and worldwide. According to the World Health Organization (WHO), suicide accounts for 800,000 deaths per year globally, with an unequal distribution between sexes, affecting three men for each women^1^. Suicides are also the second main cause of death in the population 15–29 years old. In 2016, the global age-standardized mortality rate for suicide was 11 cases per 100,000 individuals^2^. This ratio varied greatly, from three to 33 deaths per 100,000 individuals, affecting low-and-middle-income countries more^2^. In Brazil, according to the official mortality database^3^, the mortality rates for suicide totaled 6.1 deaths per 100,000 individuals in 2018^3^. The distribution between sexes followed the global trend, with a 9.7 mortality ratio per 100,000 males and 2.6 in 100,000 females.

Suicide is a preventable cause of death and multiple social and individual aspects influence this complex phenomenon^4^. Causes for people to consider and commit suicide include gender-based violence, history of childhood violence, physical, financial, and psychological violence, feelings of guilt and failure, helplessness, hopelessness, incapacity of asking for help, social isolation, lack of autonomy, functional dependency, visual deficiencies, terminal illness, depression, anxiety, suicidal ideation, psychiatric disorders, and substance abuse^5 , 6^.

Further understanding factors that influence suicide can provide important information to public authorities, health professionals, and society as a whole. Based on these factors, policies and actions can be developed to promote preventive measures against suicide. This study thus aims to investigate some of the risk and protective factors related to suicide in a large urban center in Brazil, increasing understanding of this phenomenon.

## METHODS

This is a population-based case-control study conducted in Campinas, a city located about 96 kilometers from São Paulo, the capital city of the state of São Paulo. In 2019, Campinas had around 1,167,192 inhabitants^7^, being the 3^rd^ most populous city in the state of São Paulo and 14^th^ most populous in Brazil. Campinas is a metropolis and the main city of the Metropolitan Region of Campinas, in Southeast Brazil. It constitutes an industrial and technological pole with a high human development index (HDI) (0.805), which reflects the non-pacific coexistence between wealth and poorness in large Brazilian cities which had a Gini Index of 0.56 in 2010^8^. This article is part of a broader research investigation, which analyzed the risk factors of all deaths from violent causes in 2019^9^.

### Case Sampling

The Campinas Health Department receives, from multiple sources, the totality of Death Certificates under the city’s jurisdiction. These certificates are revised and classified according to the 10^th^ revision of the International Statistical Classification of Diseases and Related Health Problems (ICD-10)^10^.

In a research partnership with the local Health Department, our study group received a database containing the information in the Death Certificates of all inhabitants of Campinas who died of external causes of death (Chapter XX in ICD-10) in any part of the national territory between January 1^st^ 2019 and December 31^st^ 2019.

From the information obtained in the Death Certificates, one of the group’s researchers contacted the family of the deceased. The research explained the aims of the research and presented an informed consent form to a member of the household (family or close relationship over 18 years old). If they accepted to participate, they were asked to sign the informed consent and underwent a structured interview. The interview was designed to obtain information (described below) about the deceased and to understand the circumstances of the death, using the method called Verbal Autopsy^11^. This study considers all those who died of suicide (X60.0 and X84.9 from the ICD-10) in 2019 in Campinas and whose families or close relationships agreed to participate.

### Control Sampling

As the case sampling method, controls were selected considering the population of Campinas. A sample of 800 random addresses from households in Campinas was obtained by a partnership protocol with Campinas’ Water Supply and Sanitation Society, which covers 99.81% of households.

Before the *in loco* application of the survey (described on Data Collection), all participants received an explanatory letter describing the research and an informed consent form. The interviewer then visited the households and explained the purpose of the visit. One out of the over-10-years-old dwellers were drawn. The aims of the research were once more explained to the drawn over-10-years-old dweller. The survey was applied after participants signed the informed consent form. Refusals were excluded from the control sampling.

### Data Collection

Data were collected from two sources. Some of the information was obtained from the Death Certificates whereas additional and complementary information was obtained by trained interviewers using a structured questionnaire. Participants classified as controls answered the same questionnaire as relatives of the deceased considering the previous 30 days. The data were gathered and classified as follows:

- Sociodemographic variables: sex (male or female), age range (10 to 29, 30 to 49, 50 to 64, 65 or more), years of schooling, paid work (yes or no), form of employment (formal or informal).
- Variables related to life events or behavioral aspects: fear of suffering violence by criminals and/or law enforcement personnel (yes or no); the presence of physical, visual, auditory, or intellectual disabilities (yes or no); use of alcohol, tobacco, marijuana, or cocaine (“do not use”, “recreational use”, “substance use disorder”); financial debt due to illicit drug purchase (yes or no); having suffered threats to one’s physical or mental integrity (yes or no).

Data gathered during the interviews were later associated with the municipal Human Development Index by Units. The Human Development Index by Units (HDIU) is a method which estimates HDI for smaller units within a metropolis according to socio-environmental characteristics^12^. In 2013, IPEA (Institute for Applied Economic Research) estimated HDIU for all metropolitan regions in Brazil^13^, reflecting more accurately sociodemographic disparities within larger political units. Similarly to the traditional HDI, HDIU is measured as a number between 0 (lower HDI) and 1 (higher HDI) and depicts economical, educational, and sanitary variables (income, schooling, and longevity)^14^. Campinas was divided into 187 Development Units in 2013. Its HDIU varied from 0.636 to 0.954.

### Analysis

The data were analyzed according to the backward method^15^. Univariate logistic regression models were initially adjusted, having each of the aforementioned variables as predictor variables and as a response to the individual’s status (case or control).

A multiple logistic regression model was then adjusted, having the sociodemographic variables as predictor variables and as a response to the individual’s status. As an entry criterion in the model, a p-value of ≤ 0.25 was adopted in the univariate analysis. To remain in the multiple models, a p-value of ≤ 0.05 was adopted.

The variables related to life events and behavioral aspects were then added to the model obtained in the previous step, using the same criteria.

Finally, in the final model, the variable HDIU was added to the adjustment obtained until then.

This research was submitted to the Research Ethics Committee of Faculdade de Ciências Médicas – Unicamp and accepted under the protocol 3.175.939, CAAE 04005118.9.0000.5404. All participants signed an informed consent form.

## RESULTS

According to the Health Department of Campinas, 606 inhabitants died in 2019 due to external causes of death. Amongst these deaths, 86 were by suicide. Seven of those suicides were not included in this research either because the families couldn’t be reached or refused to participate. After conducting and interpreting the verbal autopsies, five of the 79 remaining suicides were re-classified as having another kind of external cause of death. In turn, verbal autopsy re-classified as suicide nine deaths previously classified as an external cause of death other than suicide (two assaults, one traffic accident, one death by immolation, one exogenous intoxication, one death by drowning, and three deaths of undetermined intent). This study thus considered 83 suicide cases in total.

Regarding controls, 29 of the 800 randomly drawn households were discarded for different reasons: refusal by the potential participant; absence of the potential participant; denied access by security personnel due to condominium security policies. Of the remaining 771 households, 55 were discarded due to age policy (only people over 10 years old were interviewed). The control group thus included 716 participants.

Table 1 shows the distribution of the sociodemographic variables and the variables related to life events and behavioral aspects evaluated by verbal autopsy both for cases and controls. Table 2 shows the statistical analysis obtained in the analysis of adjusted univariate logistic regression in step 1.

**Table 1:** Spread of the sociodemographic variables and variables associated with life events and behavioral aspects evaluated.

Variable/Category	Case		Control	
	
	Abs. freq. (n)	Relat. freq. (%)	Abs. freq. (n)	Relat. freq. (%)
**Sociodemographic variables**				

Age range (years)				
10–29	32	38.6	171	23.9
30–49	27	32.5	185	25.8
50–64	17	20.5	193	27.0
≥ 65	7	8.4	167	23.3
Racial group				
White/Asian	49	59.0	473	66.1
Mixed-race	29	34.9	184	25.7
Black	5	6.0	59	8.2
Sex				
Female	19	22.9	403	56.3
Male	64	77.1	313	43.7
Marital status				
Married/civil union	32	38.6	341	47.6
Divorced/separated	14	16.9	70	9.8
Single	34	41.0	240	33.5
Widowed	3	3.6	65	9.1
Employment status				
Formal	17	20.5	273	38.1
Informal	17	20.5	104	14.5
No paid work	49	59.0	339	47.3

**Life events and behavioral aspects**				

Alcohol				
Do not use	25	30.1	447	62.4
Recreational use	34	41.0	262	36.6
Harmful use	24	28.9	7	1.0
Tobacco				
Do not use	55	66.3	612	85.5
Recreational use	17	20.5	78	10.9
Harmful use	11	13.3	26	3.6
Marijuana				
Do not use	68	81.9	687	95.9
Recreational use	6	7.2	26	3.6
Harmful use	9	10.8	3	0.4
Cocaine				
Do not use	69	83.1	710	99.2
Recreational use	2	2.4	3	0.4
Harmful use	12	14.5	3	0.4
Threats				
No	76	91.6	680	95.0
Yes	7	8.4	36	5.0
Fear^a^				
No	78	94.0	593	82.8
Yes	5	6.0	123	17.2
Criminal activities				
No	81	97.6	703	98.2
Yes	2	2.4	13	1.8
Witness of violence				
No	68	81.9	608	84.9
Yes	15	18.1	108	15.1
Disabilities				
No	43	51.8	489	68.3
Yes	40	48.2	227	31.7

**Socio-environmental variable**				

	**Mean**	**SD**	**Mean**	**SD**
HDIU^b^	0.79	0.13	0.82	0.09

Abs. freq.: absolute frequence; relat. freq.: relative frequence; SD: standard deviation; HDIU: Human Development Index by Units.

^a ^Fear of suffering violence by criminals and/or law enforcement personnel;

**Table 2:** Statistics obtained by the univariate logistic regression model.

Variable/Category	β^a^	OR	p
**Sociodemographic variables**			

Age range			
10–29 years	1.50	4.46	0.001
30–49 years	1.25	3.48	0.004
50–64 years	0.74	2.10	0.107
≥ 65	-	1	
Racial group			
White/Asian	-	1	-
Mixed-race	0.42	1.52	0.093
Black	-0.20	0.82	0.682
Sex			
Female	-	1	-
Male	1.47	4.34	< 0.001
Marital status			
Married/civil union	-	1	-
Divorced/separated	0.79	2.19	0.024
Single	0.41	1.51	0.114
Widowed	-0.71	0.49	0.251
Employment status			
Formal	-	1	-
Informal	0.97	2.63	0.010
No paid work	0.86	2.37	0.004

**Life events and behavioral aspects**			

Alcohol			
Do not use	-	1	-
Recreational use	0.82	2.27	0.006
Harmful use	4.11	60.82	< 0.001
Tobacco			
Do not use	-	1	-
Recreational use	0.91	2.47	0.006
Harmful use	1.55	4.71	< 0.001
Marijuana			
Do not use	-	1	-
Recreational use	0.86		0.091
Harmful use	3.76		< 0.001
Cocaine			
Do not use	-	1	-
Recreational use	1.44	4.23	0.215
Harmful use	3.74	42.26	< 0.001
Threats			
No	-	1	-
Yes	0.60	1.83	0.190
Fear^b^			
No	-	1	-
Yes	-1.19	0.31	0.024
Criminal activities			
No	-	1	-
Yes	0.56	1.75	0.470
Witness of violence			
No	-	1	-
Yes	0.23	1.26	0.512
Disabilities			
No	-	1	-
Yes	0.71	2.04	0.004

**Socio-environmental variable**			

HDIU			
Continuous variable	-2.84	0.06	0.015

OR: odds ratio; HDIU: Human Development Index by Units.

^a ^Beta coefficient.

^b ^Fear of suffering violence by criminals and/or law enforcement personnel.

Table 3 synthesizes the statistical analysis obtained by the multivariable logistic regression in steps 3 and 4. Fear of suffering violence by criminals and/or law enforcement personnel as well as living in high HDI neighborhoods were identified as protective factors for suicide. Fearing suffering violence was associated with a 80% suicide risk reduction. Each 0.01 increase in HDI represented a suicide risk reduction of around 4%. Moreover, being under 65 years old, male, with no paid work, using alcohol, having cocaine use disorder, and having disabilities represented risk factors for suicide, though in different magnitudes.

**Table 3:** Statistics obtained in the adjusted multivariable model.

Variable/Category	β^a^	OR	p
**Sociodemographic variables**			

Age range (years)			
10–29	1.77	5.88	0.002
30–49	1.23	3.40	0.057
50–64	1.15	3.17	0.051
≥ 65	-	1	
Sex			
Female	-	1	-
Male	1.66	5.26	< 0.001
Employment status			
Formal	-	1	-
Informal	0.76	2.14	0.130
No paid work	1.12	3.06	< 0.013

**Life events and behavioral aspects**			

Alcohol			
Do not use	-	1	-
Recreational use	0.76	2.14	0.031
Harmful use	3.50	33.12	< 0.001
Cocaine			
Do not use	-	1	-
Recreational use	-0.21	0.81	0.885
Harmful use	2.68	14.59	0.007
Fear^b^			
No	-	1	-
Yes	-1.64	0.19	0.015
Disabilities			
No	-	1	-
Yes	1.31	372	< 0.001
**Socio-environmental variable**			
HDIU	-4.05	0.02	0.008

OR: odds ratio; HDIU: Human Development Index by Units.

^a ^Beta coefficient.

^b ^Fear of suffering violence by criminals and/or law enforcement personnel.

As expected, the fourth and final step of the multivariable logistic regression analysis did not change the statistical significance of the variables evaluated in the previous steps.

## DISCUSSION

Although in recent decades suicide has been acknowledged worldwide as one of the most important global challenges in public health, the Brazilian mitigation policy against suicide was launched only in 2019, under the Federal Law 13,819, of April 26. This legislation institutes the National Policy for the Prevention of Self-Mutilation and Suicide, which among other objectives, aims to develop control actions for the determinant and conditioning factors of this preventable cause of death. However, academic research on these control actions is still incipient. Investigating the sociodemographic factors, behavioral aspects, and life events related to suicide can thus provide valuable information to develop more effective public policies and preventive actions for this cause of death.

Regarding sociodemographic conditions, the significant outcome differences between sexes, age range, and paid work stand out. Men had a higher tendency of unfavorable outcomes, corroborating national and international scientific literature. In 2010, the suicide mortality rates in the state of São Paulo were 7.5 per 100,000 inhabitants in males and 4.6 in females^16^. This difference is also observed in the Brazilian population. In a recent report about suicide in adults, the Royal College of Psychiatrists (UK) showed that three out of four people who die of suicide are male. Furthermore, suicide was the major cause of death in males under 50 years old^17^.

Regarding age range, suicides represent one of the most important causes of death in both absolute and relative values^18^. In Brazil, the latest national epidemiological report on suicide and suicidal attempts^19^ show that, whilst the suicide mortality rate was 5.5 per 100,000 inhabitants nationwide, it reached 8.9 per 100,000 inhabitants amongst people over 80 years old. Nevertheless, in Campinas, our study showed that individuals under 29 years old have a higher risk of suicide, which follows a global tendency^18^. Suicide represents 8.5% of the total causes of death in people between 15 and 29 years old and is the second main cause of death worldwide, behind only traffic accidents in the same age range^18^.

In our research, not having paid work also showed an unfavorable outcome, corroborating the international trend which indicates higher suicide risks associated with financial instability^20^. According to a 2008 multicenter research conducted in 54 countries under economic crisis, losing one’s job, foreclosure, and financial uncertainty were considered risk factors for suicide, especially when associated with other individual risk factors such as depression, anxiety, exposure to violence, and harmful use of alcohol^21^.

Importantly, the association between harmful use of alcohol and other psychoactive addictive drugs and suicide is also globally known^18^. In this sense, our research also corroborates the international literature, showing higher risks of suicide amongst people who presented recreational and harmful use of alcohol and harmful use of cocaine. A literature review with results from ten countries found the use of alcohol and other addictive substances in 25 to 50% of those who died of suicide^22^.

Although other substances such as marijuana, heroin, and nicotine are also associated with higher suicide risk^23^, our study found no statistical significance between marijuana and nicotine and suicide. In the social field, the results of our study also indicate an association between suicide and the presence of some physical/visual/auditive/intellectual disability, the HDIU of the HDI of where the cases lived, and the fear of suffering violence by criminals and/or police officers.

Having physical, visual, hearing, and cognitive disabilities were risk factors to suicide. A literature review showed triggers to suicide in people living with disabilities, such as frustration, lack of autonomy, lower sense of utility and dignity, and lower pleasure for life. In this sense, women require special attention since they frequently report refusing to overload others^24^. Moreover, some studies also show an association between chronic pain and neurodevelopmental impairment and suicide^25^.

Our study found that higher Human Development Indexes are a protective factor against suicide. In numbers, each 0.01 increase in HDI represented a 4% suicide risk reduction. Accordingly, lower income, unemployment, and poverty are risk factors for suicide globally^26^. In Brazil, other researchers also show an inverse relationship between per capita income and suicide rates^27^. Therefore, suicide must be increasingly understood as a public health, social, and economic problem.

One of the strengths of this study was the possibility of identifying how socioeconomic inequalities affect suicides. Since HDI considers health, educational, and economic conditions, this indicator reflects holistic analysis more accurately than pure economic indicators and better understands person-centered human development^8^.

Although the scientific literature indicates violence as a risk factor for suicide^28 , 29^, our research found no significant statistical difference between suicide and those who suffered domestic violence nor those who suffered threats to their physical or mental integrity. In turn, fear of suffering violence by criminals and/or law enforcement personnel were positively associated with the outcome. Fear can therefore reflect a sense of life appreciation, as opposed to hopelessness, frequently associated with suicidal behavior and suicide^30 , 31^.

Furthermore, central wealthy territories, with higher HDIU levels, presented lower rates of violence both by criminals and by abusive action of law enforcement.

Importantly, suicide is a highly complex social phenomenon which cannot be explained by a single factor. As an example, though South Korea is one of the richest countries worldwide, with low inequality levels, it presents the second-highest suicide mortality rates globally, rising from 8.8 to 33.3 deaths per 100,000 inhabitants from 1990 to 2011^31^.

Overall, our research endorses WHO’s recommendations that to create effective preventive measures against suicide, joint collaborative strategies must include multiple sectors from different governmental and non-governmental levels^18^. Therefore, a responsible broad dialogue, which involves society and media, must be encouraged. Surveillance strategies must also be reinforced, focusing on preventive policies related to mental health and psychoactive substances use.

Furthermore, health professionals’ capacitation to evaluate and treat mental health issues is essential to prevent suicide^18^. Research indicates that several people who died by suicide contacted a primary health care institution within the last 30 days before passing away^32^. In the UK, 28% of the individuals who committed suicide visited a mental health service the year of their death^33^. This shows the importance of developing a net-like health structure to deal with mental health issues in all of its aspects.

During our research, nine of the deaths registered as having violent causes other than suicide were reclassified as suicides. This indicates an underreporting of 10.8% of suicides in Campinas in 2019. This corroborates the results of a systematic review that covered 31 studies conducted in North America, Europe, Asia, and Oceania^34^, showing that 52% of the reviewed articles indicated over 10% of suicide underreporting.

As a study limitation, information regarding risk factors exposition was gathered retrospectively, which could lead to memory biases^35^. To control memory bias, interviews were conducted preferably within 15 days after the death by trained interviewers with a standard questionnaire. One of the main gaps associated with memory bias was the lack of information concerning visits to health facilities. This information would allow understanding the importance of health services in suicide prevention. This subject thus requires further studies.

Another limitation of this study is that it associated cases and controls living in the same micro area of the city with the HDIU value of the area. Depending on the social heterogeneity within the area, this may have caused misclassification, a phenomenon known as ecological fallacy. In turn, the micro areas of analysis were built preserving similar socio-environmental characteristics, which minimizes this possibility.

## CONCLUSION

Being male, aged between 10 to 29 years, without paid work, using alcohol, and presenting harmful use of cocaine were risk factors for suicide in this research. Furthermore, higher HDI levels and perception of fear had a statistically relevant protective association. These findings emphasize the multifactorial complex nature of suicide which shows that, besides individual clinical and psychological approaches, social and economic improvement policies are essential to prevent this cause of death.
